# Valganciclovir Therapy Prevents Human Cytomegalovirus Reactivation in Glioblastoma Patients Undergoing Radiochemotherapy and Extends Time to Tumor Progression

**DOI:** 10.3390/cancers18101575

**Published:** 2026-05-12

**Authors:** Mattia Russel Pantalone, Giuseppe Stragliotto, Nerea Martin-Almazan, Inti Peredo-Harvey, Jorge L. Jimenez-Macias, Afsar Rahbar, Sean Lawler, Jiri Bartek, Cecilia Söderberg-Naucler

**Affiliations:** 1 Department of Neurosurgery, Karolinska University Hospital, 17177 Stockholm, Sweden; inti.peredo@regionstockholm.se; 2 Department of Medicine, Karolinska Institute, 17164 Stockholm, Sweden; martn12@mskcc.org (N.M.-A.); afsar.rahbar@ki.se (A.R.); 3 Department of Neurosurgery, Linköping University Hospital, 58185 Linköping, Sweden; 4 Department of Neurology, Karolinska University Hospital, 17177 Stockholm, Sweden; giuseppe.stragliotto@regionstockholm.se; 5 Department of Neurosurgery, The Warren Alpert Medical School, Brown University, Providence, RI 02903, USA; jorge.luis.jimenez.macias@emory.edu (J.L.J.-M.); sean_lawler@brown.edu (S.L.); 6 Legorreta Cancer Center, Brown University, Providence, RI 02903, USA; 7 Danish Cancer Society Research Center, 2100 Copenhagen, Denmark; jb@cancer.dk; 8 Division of Genome Biology, Department of Medical Biochemistry and Biophysics, Science for Life Laboratory, Karolinska Institute, 17165 Stockholm, Sweden; 9 Institute of Molecular Genetics, The Czech Academy of Sciences, 142 20 Prague, Czech Republic; 10 Department of Biosciences, InFLAMES Research Flagship Center, MediCity, University of Turku, 20520 Turku, Finland

**Keywords:** glioblastoma, cytomegalovirus, valganciclovir, radiotherapy, chemotherapy

## Abstract

**Background:** Emerging evidence suggests that antiviral treatment targeting human cytomegalovirus (HCMV) may improve outcomes in patients with glioblastoma (GBM). In this study, we analyzed serological data from the placebo-controlled VIGAS1 trial (Eudra number 2006-002022-29), which assessed the effect of valganciclovir (VGCV) on GBM progression in 42 patients, for impact of VGCV in preventing HCMV reactivation. **Methods**: VIGAS1 patients had undergone radical surgery and were randomized to receive either VGCV (n = 22) or placebo (n = 20) alongside standard radiochemotherapy. Blood was prospectively collected at baseline and 3-, 12- and 24-week follow-up visits. GBM cell lines and a cytomegalovirus-infected murine brain cancer model were used to validate the clinical findings. **Results**: Over the 24-week study period, we found that HCMV reactivation, as inferred from IgM seropositivity, occurred in 58.3% of patients in the placebo group, whereas this was completely prevented in the VGCV-treated group except for one patient with no treatment compliance (*p* = 0.0005). HCMV reactivation was linked to early recurrence. IgG-positive patients treated with VGCV showed a significantly longer time to progression (TTP) than those receiving placebo (6.7 vs. 3.7 months, *p* = 0.0408). We found a significant association between higher steroid doses and enhanced reactivation in the placebo group. In vitro and murine studies confirmed that corticosteroids, combined with radiation therapy, enhanced cytomegalovirus reactivation, which was mitigated by antiviral treatment. **Conclusions**: These findings suggest that preventing HCMV reactivation with antiviral therapy may improve patient outcomes, especially in HCMV-seropositive GBM patients, and further support the hypothesis that HCMV is a tumor-promoting virus.

## Simple Summary

Glioblastoma is the most aggressive form of brain cancer and remains difficult to treat despite surgery, radiation, and chemotherapy. Human cytomegalovirus (a common virus carried by many adults) has been detected in glioblastoma tumors, but its role during treatment is not fully understood. In this study, we analyzed blood samples from patients undergoing radiochemotherapy and found that the virus can become reactivated during treatment, particularly in patients receiving corticosteroids. This reactivation was associated with earlier tumor recurrence. Importantly, patients treated with the antiviral drug valganciclovir did not show viral reactivation. Laboratory experiments in cell and murine models confirmed that radiation and steroids can trigger viral activity, while antiviral treatment can prevent it. These findings suggest that viral reactivation upon radiochemotherapy may contribute to disease progression and could represent a potential target for future therapeutic strategies.

## 1. Introduction

Glioblastoma (GBM) is the most prevalent and aggressive brain tumor in adults. Despite optimal treatment with surgery followed by adjuvant radiochemotherapy [1], the prognosis remains poor, with a median overall survival (OS) of 12–15 months.

Like GBM, other primary brain tumors and metastatic brain lesions are frequently treated with adjuvant radiotherapy. Although radiation remains a cornerstone of neuro-oncology, it is associated with significant side effects, including cognitive dysfunction, memory impairment, and other neurological deficits, especially when patients receive whole-brain radiation. Mechanisms underlying these adverse effects are still debated but they seem to be dose-dependent, and certain patient groups, such as the elderly patients or smokers, are more vulnerable [2,3]. Recently, evidence has emerged that reactivation of human cytomegalovirus (HCMV) following radiation therapy is frequent and might contribute to symptoms resembling radiation-induced encephalitis [4,5,6] and symptoms of radiation intolerance.

Reactivation of latent viruses, including HCMV, in immunocompromised individuals such as transplant recipients and HIV infected individuals is well-documented. Increasing evidence also demonstrates the frequent presence of an active HCMV infection in GBM and brain metastasis [7,8,9] and HCMV strains have recently been isolated from GBM [10,11]. After a primary infection, HCMV establishes latency in CD34^+^ hematopoietic stem cells in the bone marrow [12]. The virus persists in these cells for the whole life of the infected individual in myeloid lineage cells [13]. Reactivation is typically mediated by inflammatory responses [14], leading to the differentiation of these cells into pro-inflammatory macrophages or dendritic cells [15], and this occurs in 60–80% of transplant patients if no prophylactic measures are taken [16]. Furthermore, stress and DNA damage can also trigger reactivation of latent HCMV [17]. This is particularly relevant for patients with GBM, as: (i) the DNA damage response (DDR) induced by radiomimetic drugs can activate the HCMV major immediate early (IE) promoter in human GBM models via stress-induced host-cell transcription factors; (ii) HCMV expression was enhanced after radiochemotherapy in clinical GBM specimens collected at recurrency (compared with the matched pre-treatment levels in primary GBM tumors from the same patients); and (iii) cytotoxicity of experimental ganciclovir treatment correlated with HCMV presence in human GBM cells [18].

Cancer cells use sophisticated mechanisms to evade immune detection and suppress host immunity [19] and therapeutic modalities like chemotherapy and radiotherapy often exacerbate this immunosuppression, further impairing anti-viral immunity and facilitating reactivation of latent viruses such as HCMV.

After a primary HCMV infection, serological and cellular memory are established with the production of specific HCMV-reactive immunoglobulins. IgG-positivity indicates past infection, while IgM-positivity indicates recent infection or reactivation [20]. Interestingly, HCMV serological status has been linked to survival of patients with GBM, with IgG-negative patients surviving longer than IgG-positive patients [21]. The GLIO-HCMV-01 study by Goerig et al. [4] found that 48% of patients with brain tumors or metastases experienced HCMV reactivation during treatment, with neurological symptoms consistent with HCMV encephalitis. Older age and corticosteroid use were identified as risk factors. In a larger follow-up study, the same authors showed that patients who experienced HCMV reactivation and encephalopathy had a shorter OS, and that antiviral therapy with valganciclovir (VGCV) could mitigate this effect [5]. Similarly, Ursu et al. found that a quarter of IgG-positive patients with GBM developed viremia during treatment. HCMV reactivation was linked to older age, steroid intake, and low lymphocyte counts before radiotherapy. While neurological symptoms were evident, progression-free survival was not significantly affected [6]. Other studies have also reported HCMV reactivation in cancer patients treated with temozolomide and steroids, speculating that the immune suppression caused by these drugs might facilitate reactivation [22,23,24,25].

Over the last two decades, HCMV has emerged as a potential important oncomodulatory factor in GBM and other human cancers, as this virus is able to influence all ten hallmarks of cancer [26]. Keeping HCMV at bay in patients with GBM may therefore impact patient outcome. In further support of this statement, our research has shown that high levels of HCMV in GBM tumors are associated with shorter survival [27,28]. We also found that HCMV appears to be correlated with focal-to-bilateral seizures in patients with tumor-related epilepsy, further correlating with shorter survival [29].

In immunocompromised individuals, antiviral prophylaxis or pre-emptive therapy is standard for managing HCMV [30]. VGCV, a nucleoside analogue, is the primary antiviral agent used both for treatment and prophylaxis [31]. In 2006, we initiated the VIGAS trial, the first attempt to treat GBM patients with VGCV [32]. This pilot study aimed to evaluate the drug’s safety and potential efficacy in preventing tumor progression and extending survival, providing information to guide the design of subsequent clinical trials. As expected, the VIGAS study was underpowered and did not meet its primary endpoint, but it showed trends toward reduced tumor growth. More importantly, exploratory analyses indicated the survival rate in VGCV-treated patients was significantly higher. Follow-up retrospective analyses of patients treated with VGCV as an add-on to first- or second-line therapy confirmed these findings in primary, [33] recurrent [34] and secondary GBM [35]. We are currently performing a prospective double-blind, randomized clinical trial, VIGAS2, in 220 patients with primary GBM to confirm or refute these promising findings. Recruitment is completed and study results are expected in end of 2027.

During the initial VIGAS1 trial (NCT00400322), blood samples were collected at multiple timepoints to monitor HCMV serostatus. Given recent insights on the role of HCMV reactivation and its possible impact on tumor progression, we performed new analyses on VIGAS1 patients in the context of concurrent radiochemotherapy. We report here that HCMV reactivation is common following chemoradiation in GBM patients and is associated with shorter time to progression. Importantly, VGCV therapy appeared to prevent reactivation and improve clinical outcomes, findings that were corroborated in both in vitro and in vivo models.

## 2. Materials and Methods

### 2.1. Study Design

This study included all the patients who participated in the VIGAS1 study. The study was registered at the Swedish medical agency (Eudra number 2006-002022-29) and at ClinicalTrials.gov (Identifier NCT00400322), and was approved by the Karolinska ethics committee (2006/755-31). Additional studies on patients receiving VGCV were also approved by the regional ethics committee in Stockholm (Dnr: 2016/1426-31/1). The inclusion and exclusion criteria, as well as patient details, were previously described [32]. Data relevant to the clinical trial were collected from the deposited database, including dates of the start and end of radiochemotherapy, details on clinical status, corticosteroid therapy, and any additional relevant information derived from patients’ records. Raw data from serological analyses were retrieved from the study database, and new serological tests were re-run for samples previously omitted or classified as “gray zone” or “unsure” using the HCMV serology kit from Abcam, following the manufacturer’s instructions.

### 2.2. In Vitro Treatment

U251 (ECACC 09063001) and U373 (Uppsala, ECACC 08061901) cell lines were cultured in RPMI-1640 (Thermo Fisher Scientific, Inc.. Waltham, MA, USA) supplemented with 10% fetal bovine serum (Sigma-Aldrich; Merck KGaA Darmstadt, Germany) and 100 U/mL penicillin and streptomycin at 37 °C in 5% CO_2_/95% air. Approximately 1.5 × 10^5^ U251 and U373 cells were plated in 6-well plates in the presence or absence of 100 μM ganciclovir (GCV; Roche Diagnostics, Mannheim, Germany) and/or Betamethasone (100 nM, Sigma-Aldrich; Merck KGaA) for 24 h at 37 °C, then infected with HCMV strain VR1814 at a multiplicity of infection (MOI) of 3, or mock-infected. At 1-, 3-, and 7-days post-infection (dpi), the cells were lysed and collected using TRIzol (Thermo Fisher Scientific, Inc.). Samples were stored at −80 °C for up to 1 month. RNA extraction, reverse transcription, and TaqMan PCR analyses were performed as previously described for HCMV IE transcripts [36]. All human cell lines were authenticated by the commercial source, and all experiments were conducted using mycoplasma-free cells.

### 2.3. In Vivo Studies

Animal experiments were conducted at Brown University in Dr Lawler’s laboratory at the Legorreta Cancer Center, Brown University. The study was approved by the local committee (Protocol Number: 21-08-0005). Six-week-old male and female C57BL/6 mice were purchased from Charles River Laboratories and mated once. The F1 generation was inoculated with a nonlethal dose of Δ157 MCMV Smith strain at postnatal day 2 (P2) (10^3^ PFU) via intraperitoneal (i.p.) injection, as previously described [37]. A total of 25 mice were injected with MCMV while 10 mice were used as controls and were injected with PBS. One MCMV-infected mouse was lost because of an infection. After 14 weeks, 5000 GL261Luc2 cells in 3 μL of normal saline were injected intracranially to establish mouse brain tumors (2 mm right lateral, 1 mm frontal to the bregma, and 3 mm deep) in the remaining 34 mice (24 infected, 10 uninfected). Two weeks later, tumor growth was assessed using the Xenogen in vivo imaging system (IVIS). Mice were then randomized into the following treatment groups: untreated, radiotherapy, radiotherapy + dexamethasone, ganciclovir, and radiotherapy + dexamethasone + ganciclovir. Only mice previously infected with MCMV were treated with ganciclovir, with each group consisting of 5 mice. Ganciclovir (50 mg/kg) and dexamethasone (10 mg/kg) treatments were administered by i.p. injection 3 times per week for up to 6 weeks. DMSO and sterile saline were used as vehicle controls. Radiotherapy was delivered under isoflurane inhalational anesthesia with physiological monitoring (pneumatic cushion; breathing rate 40–60 breaths/min) on a heated mat using a Gulmay 320 irradiator (300 kV, 10 mA, 2.25 Gy/min, Gulmay Ltd., Byfleet, Surrey, UK).

qRT-PCR: Total RNA from blood was extracted using the QIAamp Viral RNA Mini Kit (QIAGEN, Hilden, Germany, Cat. No. 52906). mRNA expression analysis was carried out using Power SYBR Green (Applied Biosystems, Thermo Fisher Scientific, Waltham, MA, USA). RNA concentration was quantified using a Nanodrop RNA 6000 (Thermo Fisher) and analyzed using the 7900HT Real-Time PCR System (Cat. No. 4329001, Applied Biosystems, Thermo Fisher Scientific, Inc.). Ppia (Fwd 5′-CAAACACAAACGGTTCCCAG-3′/Rev 5′-TTCACCTTCCCAAAGACCAC-3′) primers were used as the housekeeping gene and mIE1 primers as the target gene (Fwd 5′-AGCCACCAACATTGACCACGCAC-3′/Rev 5′-GCCCCAACCAGGACACACAACTC-3′).

### 2.4. Statistical Analysis

The study evaluated patients for key survival metrics, including median time-to-tumor progression (TTP) and median OS. TTP and survival data are represented using Kaplan–Meier curves, calculated from the date of surgery. Statistical significance was determined using the log-rank test. Fisher’s exact test and the Mann–Whitney test were performed to assess the difference between the two groups. All statistical tests were two-sided, with significance defined at a 5% level (*p* < 0.05). Analyses were conducted using GraphPad Prism (version 10).

## 3. Results

### 3.1. Patient Demographics and Treatment Regimen

The VIGAS study included 42 patients with primary GBM who were randomized to receive either VGCV (n = 22) or placebo (n = 20) in a double-blind fashion. Notably, one patient from the VGCV group was excluded from survival analysis due to non-compliance with therapy. Sixteen patients in the VGCV group received full-dose radiochemotherapy while three received temozolomide only and two received only radiotherapy. Fourteen patients in the placebo group received full-dose radiochemotherapy, four temozolomide only and two radiotherapy only. The median time from first surgery to radiochemotherapy was 41 days and the length of radiotherapy was 43 days. VGCV therapy was initiated a median of 20.5 days before the commencement of adjuvant radiochemotherapy. These treatment schedules align with standard clinical practices for GBM, ensuring the study cohort was comparable to typical patient populations.

### 3.2. HCMV Reactivation and Serological Data

**Figure 1:**
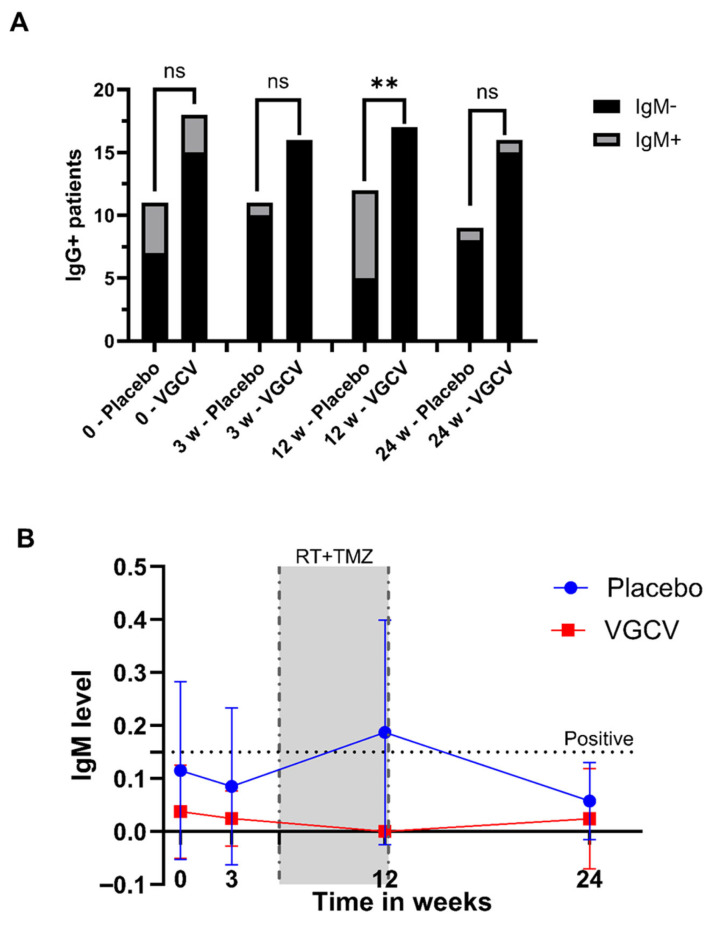
HCMV serology status of VIGAS 1 patients over time. (**A**) Bars show the number of IgG-positive patients in placebo and VGCV treatment groups over time. IgM-negative patients are shown in black while IgM-positive patients are shown in gray. There is no statistically significant difference between IgM-positive and -negative in any time point except for patients receiving placebo at 12 weeks. (**B**) The graph shows IgM levels over time for patients receiving placebo (marked in blue) and patients receiving VGCV, marked in red. The threshold for positivity is represented by a dotted line. The time interval in which patients received radiochemotherapy is depicted in light gray.

Serological data for HCMV (IgG and IgM) were collected for all patients at enrollment (baseline) and at 3, 12, and 24 weeks after inclusion. Results were crossed with therapeutic intervention and clinical data. At baseline, patients started treatment with the study drug (VGCV or placebo), and at 3 weeks follow-up patients were about to start adjuvant radiochemotherapy, which was then in late phase or completed at 12 weeks follow-up. At 24 weeks, the study ended and final follow-up was concluded. At baseline, 18 of 22 (81.8%) patients in the VGCV group and 11 of 20 (55%) in the placebo group were HCMV IgG-positive (Figure 1), indicating prior exposure to the virus. The difference in serological positivity at inclusion was not significant (Fisher’s exact test, *p* = 0.5265). At 3 weeks, two IgG-positive patients in the placebo group were IgM-positive. None of the VGCV -treated patients were IgM-positive at 3 weeks. The difference was not statistically significant (Fisher’s exact test, *p* = 0.1746).

We observed a significant difference in HCMV reactivation rates at 12 weeks between the groups who underwent radiochemotherapy: none (0/17) of the IgG-positive patients in the VGCV group developed IgM positivity (Figure 1), a result that aligns with the known antiviral effects of VGCV, which prevents reactivation and inhibits HCMV replication [38]; conversely, 58.3% (7/12) of the IgG-positive patients in the placebo group became IgM-positive by the 12-week follow-up, suggesting viral reactivation (Figure 1). The difference between IgM positivity was statistically significant at this time point (Fisher’s exact test, *p* = 0.0005). One HCMV IgG-negative patient in the placebo group became IgG-positive at the 12-week time point. As expected, all IgG-positive patients at inclusion remained positive in the subsequent follow-up analyses.

### 3.3. Time to Progression (TTP) and Survival Analysis

**Figure 2:**
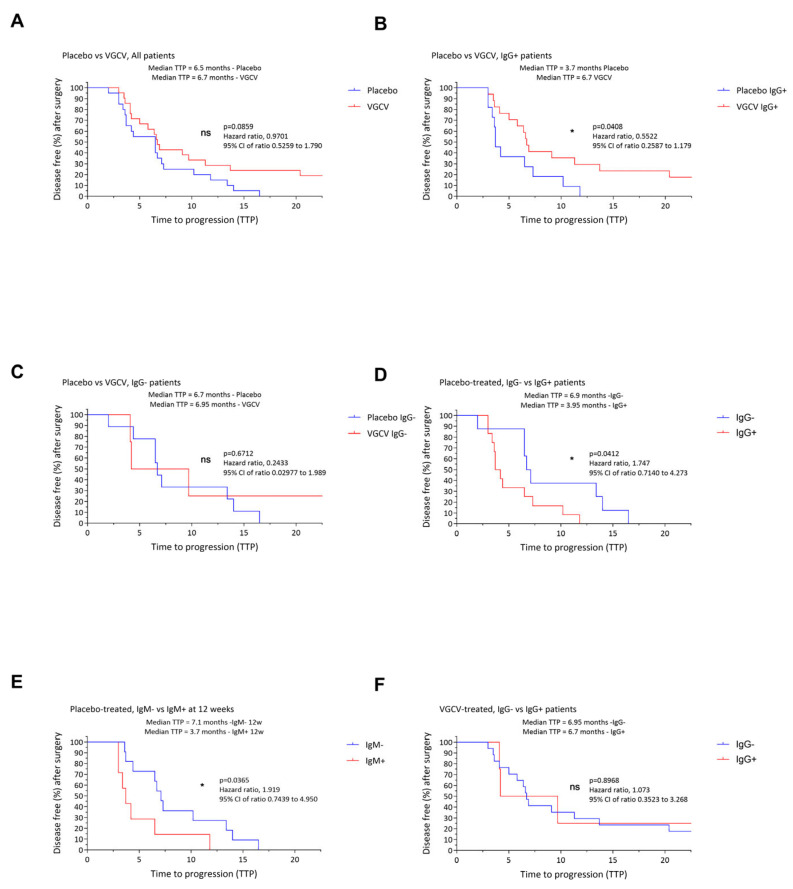
Time to tumor progression in placebo and valganciclovir-treated patients in VIGAS 1. Kaplan–Meier curves showing time to tumor progression (TTP) in months according to HCMV serological status for IgG and IgM in patients receiving placebo or valganciclovir (VGCV). Specifically in all patients receiving pacebo versus patients receiving VGCV (**A**), in IgG positive patients receiving pacebo versus patients receiving VGCV (**B**), in IgG negative patients receiving pacebo versus patients receiving VGCV (**C**), in IgG-negative versus IgG-positive placebo-treated patients (**D**), in IgM-negative versus IgM-positive placebo-treated patients at 12 weeks (**E**), in IgG-negative versus IgG-positive VGCV-treated patients (**F**). Significance is indicated as follows: ns (non-significant); * (*p* ≤ 0.05).

The impact of HCMV reactivation on TTP was next explored in both the placebo and VGCV groups. Interestingly, while no significant difference in overall TTP was observed between the VGCV and placebo groups (6.7 vs. 6.5 months, *p* = 0.0859; Figure 2A), stratification by HCMV IgG status revealed that IgG-positive patients receiving VGCV had a significantly longer TTP than those IgG-positive in the placebo group (6.7 vs. 3.7 months, *p* = 0.0408; Figure 2B). However, there was no difference in TTP among the two groups for IgG-negative patients (6.95 vs. 6.7 months, *p* = 0.6712; Figure 2C).

Overall, patients receiving placebo who were IgG-positive had shorter TTP than IgG-negative patients (3.95 months vs. 6.9 months, *p* = 0.0412, Figure 2D). This reinforces the idea that preventing HCMV reactivation through VGCV therapy can delay disease progression, particularly in a subset of patients at risk for reactivation. Indeed, the TTP for IgM-positive patients at 12 weeks was only 3.7 months as compared with 7.1 months in the IgM-negative patients that received placebo treatment (*p* = 0.0365, Figure 2E). No difference was observed in TTP in IgG-positive vs. IgG-negative patients in the VGCV group (6.7 vs. 6.95 months, *p* = 0.8968, Figure 2F), suggesting that the patients at risk for reactivation (IgG-positive) were pre-emptively protected from early recurrence by VGCV treatment.

Among the seven patients in the placebo group who showed HCMV reactivation (IgM-positive at 12 weeks), five experienced very early tumor recurrence (within 2 to 4 months), indicating a potential role for HCMV in accelerating tumor growth.

### 3.4. HCMV Reactivation and Tumor Recurrence

The role of HCMV in promoting tumor recurrence is of particular interest, as it suggests that reactivation of the virus could be a modifiable factor that affects tumor progression. According to the study design of the VIGAS1 trial, for ethical reasons, the diagnosis of recurrence allowed the patient to withdraw from the study and choose to start VGCV on a compassionate use basis, if they wished. Of the twenty patients that were randomized to receive placebo, twelve received VGCV when they exited the study, either for an early recurrence or at the end of the 6-month randomization. This is a relevant confounding factor for OS; therefore, these analyses were not included in the present study. However, it is interesting to observe that among the seven patients that were IgM-positive at 12 weeks, five of them also had an early recurrence and three of these decided to start VGCV as they exited the trial because of progressive disease. All three had an MGMT unmethylated promoter status indicating worse prognosis. Notably, the three patients who initiated VGCV therapy after tumor recurrence on compassionate use survived substantially longer (16.4, 19.5, and 55.7 months) than another patient who also had MGMT unmethylated promoter status who declined antiviral treatment and survived only 9.7 months. At 24 weeks, only one of the nine IgG-positive patients in the placebo group was IgM-positive. This patient had not been treated with VGCV after exiting the trial. One of the sixteen IgG-positive patients in the VGCV group was also IgM-positive at 24 weeks. Interestingly, this patient had discontinued VGCV treatment after the 12-week follow-up time point as the patients exited the trial due to recurrent disease at the week-24 follow-up. While these findings are promising, the retrospective nature of the compassionate use data limits the conclusions we can draw about the direct impact of VGCV on survival. Nevertheless, they point to the possibility that early intervention with antiviral therapy may be beneficial for patients experiencing HCMV reactivation and early tumor recurrence.

### 3.5. Corticosteroid Use and HCMV Reactivation

**Figure 3:**
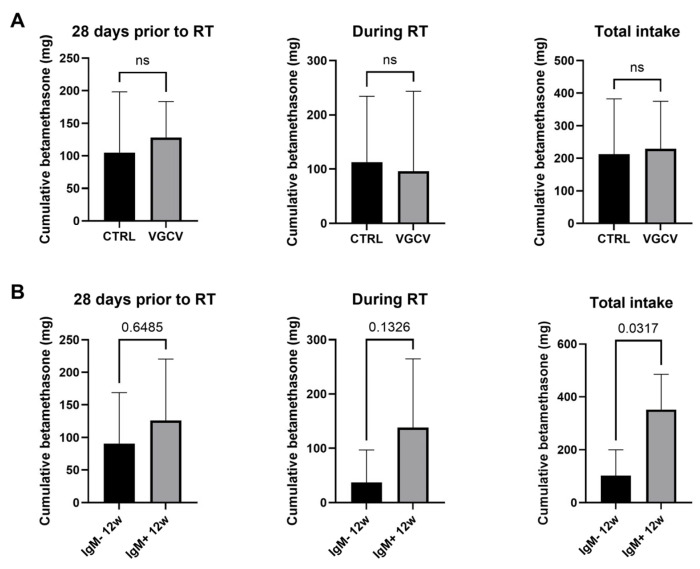
Cumulative dose of bethametasone treatment in VIGAS 1 patients. (**A**) Betamethasone amount (mg) in patients receiving placebo (CTRL) vs. valganciclovir (VGCV) before and during radiochemotherapy. (**B**) Betamethasone amount (mg) according serological status of patients at 12 weeks: IgM-positive vs. IgM-negative patients are shown. *p*-values or ns (non-significant) are indicated on top.

Corticosteroids are commonly prescribed to manage symptoms in patients with GBM, especially those undergoing radiation therapy. As corticosteroid use has been implicated in promoting HCMV reactivation and in promoting viral reactivation in brain tumors [4,6], but also in other contexts including organ transplant recipients and HIV-positive individuals [39,40], we analyzed the impact of corticosteroid treatment on HCMV reactivation. We found no difference in total steroid intake between patients in the placebo and VGCV groups, neither pre- nor during radiotherapy, as assessed by the Mann–Whitney test. However, a higher dose of total corticosteroid intake was associated with HCMV reactivation for patients in the placebo group (as assessed by HCMV IgM positivity at 12 weeks, *p* = 0.0317) (Figure 3).

**Figure 4:**
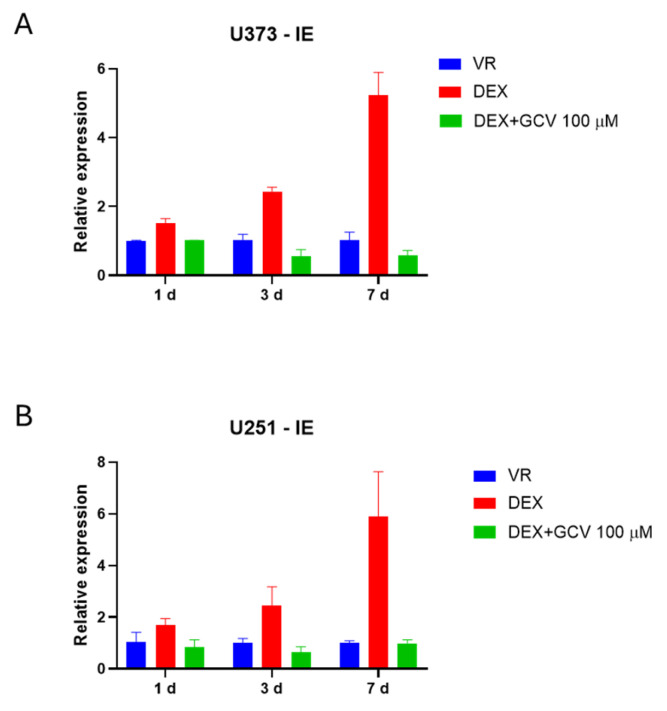
IE transcript expression U373 (**A**) and U251 HCMV-infected cells (**B**). Relative IE expression was determined by reverse transcription-quantitative PCR at 1, 3 and 7 dpi. Data are presented as the mean ± SD. Glioma cells infected with HCMV and treated with dexamethasone (DEX) show higher IE levels than untreated infected cells (VR). Additional treatment with ganciclovir (GCV) significantly reduces IE transcripts in DEX-treated cells at all time points.

In further support of this statement, in vitro studies conducted on GBM cell lines (U251 and U373) showed that dexamethasone treatment significantly increased HCMV gene expression, suggesting that corticosteroids can promote viral replication in GBM cells. GBM cell lines (U251 and U373) were cultured, infected with the HCMV strain VR1814 in the presence or absence of dexamethasone (DEX, dose 100 nM), and harvested at 1, 3 and 7 dpi. The tumor cell-derived RNA was subjected to RT-qPCR for HCMV IE transcript analyses. Mock-infected cells did not express any viral transcripts, while HCMV IE transcripts were detected in both cell lines upon infection. DEX-treated, HCMV-infected cells showed significantly higher levels of viral transcripts at all timepoints analyzed (Figure 4). Interestingly, pre-treatment with ganciclovir (100 µM for 2 h) was able to prevent this increase in viral transcripts, suggesting that antiviral therapy may mitigate the risk of HCMV reactivation in patients receiving corticosteroid therapy.

### 3.6. Corticosteroids, Radiation Therapy, and HCMV Reactivation in Murine Models

**Figure 5:**
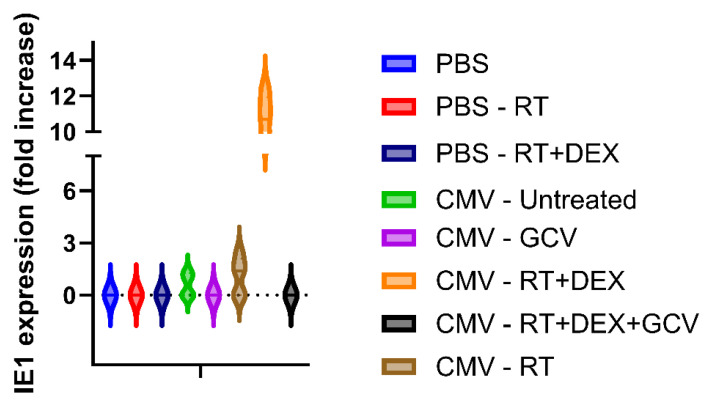
Murine IE1 expression in blood of glioma-bearing mice. PCR showing IE1 expression in mice bearing gliomas and previously infected (CMV) or not (PBS) with murine cytomegalovirus and treated with ganciclovir (GCV), radiotherapy (RT) and dexamethasone (DEX) or in combination.

To further explore the interplay between corticosteroids, radiation, and HCMV reactivation, we used a murine model of GBM. We established latent MCMV infection in twenty-five mice during 14 weeks, of which one mouse was lost. Ten mice were used as control and injected with only PBS. The mice (n = 34) were then subjected to intracranial tumor cell implantation. After two weeks, the mice were treated with ganciclovir and DEX and subjected to radiation therapy as described in Section 2. Blood was collected intracardially from mice when they were sacrificed either because they developed neurological signs or at the end of the follow-up set at 45 days. Blood samples were frozen at −80 °C and thawed for RNA extraction and cDNA synthesis. A PCR assay was run to determine transcript levels of MCMV IE. None of the uninfected mice had detectable levels of MCMV RNA transcripts in their blood, while 10/24 of the MCMV-infected mice (2/4 of the untreated, 3/5 of the RT group, and 5/5 of the RT+DEX treated group) were RNA-positive for MCMV IE in blood (Figure 5). Thus, latently infected mice treated with radiation and steroid therapy showed significantly higher levels of MCMV RNA in the blood, particularly when the two treatments were combined in comparison with untreated mice or mice treated only with radiation. Ganciclovir administration effectively prevented viral reactivation in infected and untreated mice and even in radiation- and corticosteroid-treated mice. These findings underscore the potential for corticosteroids and radiation to synergistically promote HCMV reactivation and the protective role of antiviral therapy in mitigating this effect.

## 4. Discussion

### 4.1. HCMV Reactivation and Clinical Impact in Glioblastoma

The findings of this study suggest that HCMV reactivations are common in patients with GBM after adjuvant therapy and that VGCV treatment effectively prevents HCMV reactivation in those patients. This is consistent with prior studies demonstrating a link between HCMV reactivation and neurological decline [4,5,6], as well as its potential negative impact on tumor progression [5]. In this study, we observed a striking difference between patients who received VGCV and those who did not: none of the VGCV-treated patients experienced HCMV reactivation upon adjuvant radiochemotherapy, whereas over half of the placebo group patients (58.3%) developed IgM positivity, indicative of viral reactivation. These findings underscore the potential role of antiviral prophylaxis in mitigating the negative effects of HCMV in GBM patients.

In our study, HCMV reactivation was notably associated with a significantly shorter TTP, especially for IgM-positive patients in the placebo group (median TTP 3.7 months) compared to IgM-negative patients (median TTP 7.1 months, *p* = 0.0365). These findings suggest that HCMV reactivation may accelerate tumor progression in GBM patients, a mechanism likely reflecting a direct effect of HCMV on the tumor cells as well as immune modulatory effects of the virus on the tumor microenvironment [41]. Importantly, our findings underscore the potential high effectiveness of VGCV in blocking HCMV reactivation, a key component in potentially mitigating treatment-related complications and virus-induced tumor progression in GBM patients.

The connection between HCMV reactivation and poor prognosis in patients with GBM was earlier observed by other researchers [4,5,6]. Similarly, our study supports these observations, as patients with HCMV reactivation had encephalitis-like symptoms (earlier likely considered neurological decline due to radiation-induced negative effects on the brain) and notably, these patients had a significantly shorter TTP compared to those who did not reactivate the virus. While the difference in TTP between the VGCV and placebo groups was not statistically significant at the overall level (*p* = 0.0859), we observed a significant benefit among IgG-positive patients who received VGCV, with a longer TTP (6.7 months) compared to the placebo group (3.7 months, *p* = 0.0408). These results align with previous reports suggesting that patients with a history of HCMV infection are at a higher risk for reactivation, which can exacerbate both viral and tumor-related pathologies [4,5,6]. None of the patients in the VGCV group reactivated HCMV while on VGCV therapy, and they all tolerated radiation therapy, further implying that the neurological decline in some patients after radiation therapy is caused by HCMV reactivation and virus-induced symptoms rather than by radiation therapy itself.

### 4.2. The Role of Steroid Therapy and Corticosteroids in HCMV Reactivation

Steroid therapy is a common adjunct in GBM treatment, often used to manage oedema and neurological symptoms associated with the tumor and its treatment. However, as corticosteroids have been shown to exacerbate viral reactivation in immunosuppressed patients, we considered the possibility that corticosteroids would worsen the situation for HCMV-reactivating patients. In the present study, we indeed found that higher corticosteroid use was associated with a greater risk of HCMV reactivation among placebo patients. This is consistent with the work of Ursu et al., who showed that corticosteroid use, along with a lower lymphocyte count before radiotherapy, increased the risk of HCMV reactivation in GBM patients [6]. Our in vitro experiments further corroborated this clinical observation, as DEX treatment in HCMV-infected GBM cell lines resulted in increased levels of viral transcripts, suggesting that corticosteroids can promote HCMV replication in tumor cells also independently of immunosuppression. These data are coherent with the previous published literature showing that glucocorticoids directly induce viral gene expression in latently infected cells [39,42].

The use of VGCV in this study appeared to counteract the radiation- and corticosteroid-induced reactivation of HCMV, supporting its potential role as both a preventive and therapeutic intervention in GBM. Our murine studies further corroborated the finding that radiation, particularly when combined with corticosteroid treatment, resulted in significantly higher levels of MCMV transcripts, a finding that is also in line with previous reports in patients with brain cancer [5]. Although ganciclovir was administered intermittently in the murine model, in contrast to daily VGCV dosing in patients, the experimental design aimed to achieve sufficient antiviral exposure to suppress viral replication. While pharmacokinetic differences limit direct translation, the consistent suppression of viral reactivation across models supports the biological plausibility of the clinical observations. The improved TTP observed in our study for VGCV-treated patients, particularly in those at risk for HCMV reactivation, highlights the potential for targeted antiviral strategies in this population.

Mechanistically, the radiation-induced activation of DNA-damage–response pathways might, through expression of stress-induced transcription factors, influence the expression of viral genes to drive viral reactivation [18]. Radiation therapy causes tissue inflammation and can promote the release of cytokines and growth factors that alter the tumor microenvironment. These changes can activate latent HCMV through the induction of pro-inflammatory pathways, as inflammatory responses due to radiation may trigger HCMV reactivation [14]. As radiation therapy can impair the local immune response to infections, it can facilitate further amplification of the reactivated virus. Radiation therapy can cause tumor cells to become more permissive to viral replication [18], especially if the tumor cells are already harboring latent HCMV. This could result in higher levels of viral transcripts, as shown in our in vitro and in vivo studies.

### 4.3. Potential Impact of VGCV on TTP and Survival

The reactivation of HCMV during or after radiation therapy could have important implications for patient outcomes, as HCMV already has been associated with poor prognosis in some cancers in addition to GBM, such as breast [43], colon [44] and ovarian cancer [45].

In our analysis, the impact of VGCV on survival outcomes was especially interesting. Although it was not possible to perform OS analyses because most of the patients started VGCV as compassionate therapy after the randomization period, patients who received VGCV and did not experience HCMV reactivation showed a longer TTP, particularly those with pre-treatment IgG positivity. These findings suggest that HCMV reactivation may serve as a modifiable factor influencing tumor progression, and by preventing viral reactivation, antiviral therapy could potentially improve clinical outcomes by preventing the virus’s ability to promote tumor growth. This is consistent with findings from our previous retrospective studies of patients with GBM who have received VGCV as add-on to standard therapy [33], as well as in explorative analyses in the VIGAS1 trial, which reported trends towards prolonged survival in VGCV-treated patients with GBM [32]. None of the IgG-positive patients in the VGCV group developed IgM positivity, while this was observed in 58.3% of placebo patients, a result that aligns with the known antiviral effects of ganciclovir that inhibits HCMV replication and prevents reactivation [38]. The findings underscore the effectiveness of VGCV in blocking HCMV reactivation, and thereby potentially mitigating treatment-related complications in GBM patients. Other strategies to achieve control of HCMV include vaccination strategies [46] and adoptive therapy of HCMV specific T cells [47], and these strategies are also feasible and indicate improved survival in patients with GBM [48], which further highlights the importance of controlling HCMV infection in GBM patients to improve patient outcome. Of note, dendritic cell therapy approaches using tumor cell lysates may also involve an effect of HCMV, as HCMV pp65-specific T cells have been shown to be expanded in patients treated with dendritic cell vaccination [49,50]. We therefore hypothesize that VGCV influences GBM progression by suppressing HCMV, thereby reducing the activation of oncomodulatory pathways driven by the virus within tumors and potentially also by preventing HCMV encephalopathy, although the possibility of off-target effects cannot be ruled out. This underscores the importance of timely antiviral intervention, particularly in patients who exhibit signs of HCMV reactivation after radiochemotherapy. The optimal timing of antiviral therapy remains to be determined and will be further explored in VIGAS2.

Altogether we propose that antiviral treatment is likely most beneficial as a prophylactic strategy, especially among anti-HCMV IgG-positive patients, to avoid reactivation of HCMV, instead of treating the reactivated virus once it already worsens the clinical course of the disease. HCMV-IgG testing could therefore be used as a stratification biomarker to identify patients at risk of reactivation who may benefit from pre-emptive treatment with VGCV during radiochemotherapy. However, routine screening cannot be recommended based on this study alone and should be evaluated prospectively in larger trials. In the meantime, treating physicians should remain aware of this potential association and consider it in relevant clinical contexts.

### 4.4. Study Limitations and Future Directions

While the findings from this study are promising, it is important to acknowledge that it has limitations. The VIGAS1 trial was not powered to detect differences in survival endpoints, and all efficacy analyses should therefore be interpreted as exploratory. The small sample size of the initial VIGAS trial limits the strength of the conclusions that can be drawn. Although the majority of patients received standard concomitant radiochemotherapy, a subset received temozolomide or radiotherapy alone. This reflects real-world clinical practice but introduces treatment heterogeneity, which may confound the observed associations between antiviral therapy and time to progression. Importantly, the distribution of treatment regimens was comparable between the VGCV and placebo groups, reducing but not eliminating the risk of systematic bias. Out of the seven patients that that reactivated the virus, six received combined radiochemotherapy and one received just radiotherapy but no temozolomide. Although a higher proportion of patients in the VGCV group were IgG-positive at baseline, this difference was not statistically significant and did not appear to influence reactivation rates, as reactivation was effectively prevented in the VGCV group irrespective of baseline serostatus. HCMV reactivation was primarily inferred from IgM seropositivity, which, while suggestive of recent infection or reactivation, is not as specific as direct virological assays such as CMV DNAemia or antigenemia; thus, misclassification cannot be excluded.

These findings should therefore be interpreted as hypothesis-generating and warrant confirmation in more homogeneous cohorts such as the ongoing VIGAS2 trial. However, our present data come from a double-blinded randomized clinical study where the timing of VGCV initiation and patient selection would not be subject to any bias. Future randomized controlled trials with larger cohorts, such as the ongoing VIGAS2 study, will be crucial to confirm the efficacy of VGCV in preventing HCMV reactivation and improving survival in GBM patients. Additionally, further investigation into the mechanistic pathways through which HCMV reactivation contributes to tumor progression will help elucidate the potential of antiviral therapy as an adjunct to standard treatments. Although ganciclovir was administered intermittently in the murine model, in contrast to daily VGCV dosing in patients, the experimental design aimed to achieve sufficient antiviral exposure to suppress viral replication. While pharmacokinetic differences limit direct translation, the consistent suppression of viral reactivation across models supports the biological plausibility of the clinical observations. Overall, while the murine model provides valuable insights into the mechanisms underlying HCMV reactivation, further studies in humans are needed to validate these findings and assess the potential therapeutic benefit of antiviral therapy in preventing HCMV-associated tumor progression in clinical settings.

## 5. Conclusions

This study provides compelling evidence that HCMV reactivation during radiochemotherapy is associated with a shorter time to progression in GBM patients, and that VGCV therapy can prevent HCMV reactivation and improve TTP in IgG-positive patients. By mitigating the harmful effects of viral reactivation, VGCV may help to reduce neurological decline and improve survival outcomes. Corticosteroid use, commonly prescribed in these patients, was found to promote HCMV reactivation, further supporting the potential need for antiviral strategies in this high-risk group. The findings suggest that targeted antiviral therapy, such as VGCV, may offer a promising approach to improving outcomes for GBM patients undergoing immunosuppressive, genotoxic treatments. However, further studies with larger patient cohorts are needed to confirm the long-term benefits of this strategy and to explore its potential role in combination with other therapeutic interventions.

## Data Availability

The data will be made available upon reasonable request to the corresponding author.

## Abbreviations

The following abbreviations are used in this manuscript:

HCMV	Human cytomegalovirus
GBM	Glioblastoma
VGCV	Valganciclovir
TTP	Time to tumor progression
MCMV	Murine cytomegalovirus
OS	Overall survival
DDR	DNA damage response
IE	Immediate early
i.p.	Intraperitoneal
DXM	Dexamethasone
MOI	Multiplicity of infection
GCV	Ganciclovir
